# Supplementary material for: Development and Validation of the New Liquid Chromatography-Tandem Mass Spectrometry Method for the Determination of Unbound Tacrolimus in the Plasma Ultrafiltrate of Transplant Recipients
Source: Pharmaceutics. 2022 Mar 12;14(3):632. doi: 10.3390/pharmaceutics14030632 (PMC8951301; doi:10.3390/pharmaceutics14030632)
Supplement: Supplementary file 1 [file pharmaceutics-14-00632-s001.zip › pharmaceutics-1611311.pdf]

pharmaceutics

MDPI

Supplementary Materials: Development and Validation of the New Liquid Chromatography-Tandem Mass Spectrometry Method for the Determination of Unbound Tacrolimus in the Plasma Ultrafiltrate of Transplant Recipients

Magdalena Bodnar-Broniarczyk, Karola Warzyszyńska, Katarzyna Czerwińska, Dorota Marszałek, Natalia Dziewa, Maciej Kosieradzki and Tomasz Pawiński

	Experiment		
	**1**	2	3
Solvent	**cyclohexane**	hexane	cyclohexane
Concentration [pg/mL]	**100**	100	100
Volume [μL]	**1500**	1500	1500
Protein precipitation mixture [μL]	**250**	250	none
Retention Time [s]	**1.473**	1.474	1.48
Area	**49623**	34657	15820
Signal/Noise	**66.15**	50.72	72.75
Internal Standard	**58223**	41779	19957

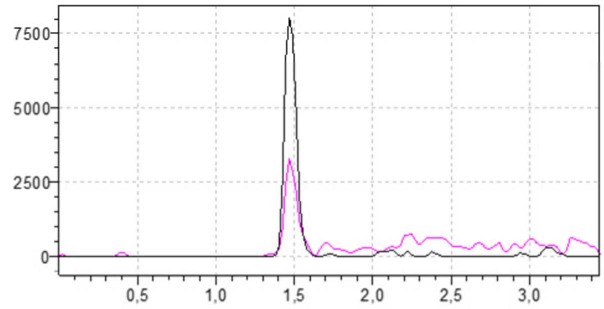

(1)*

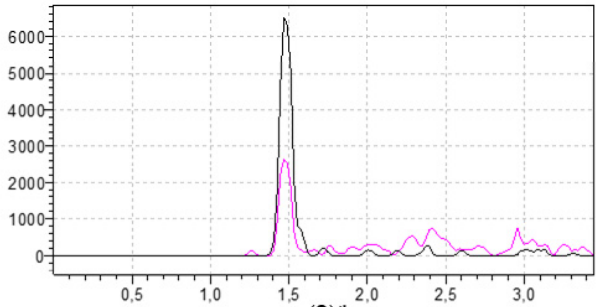

(2)*

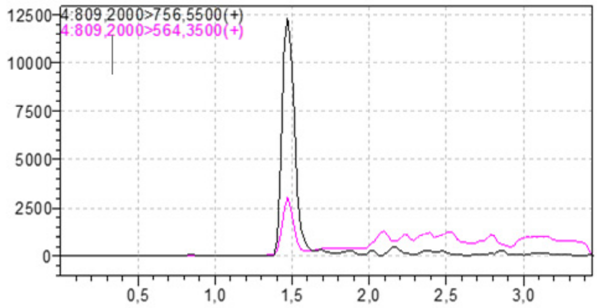

(1)**

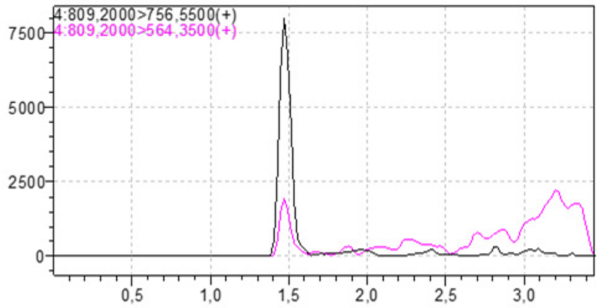

(2)**

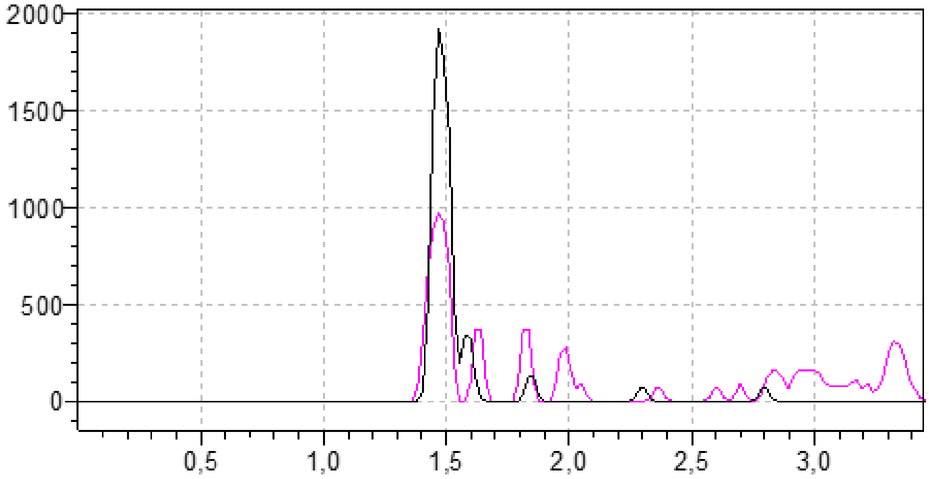

(3)*

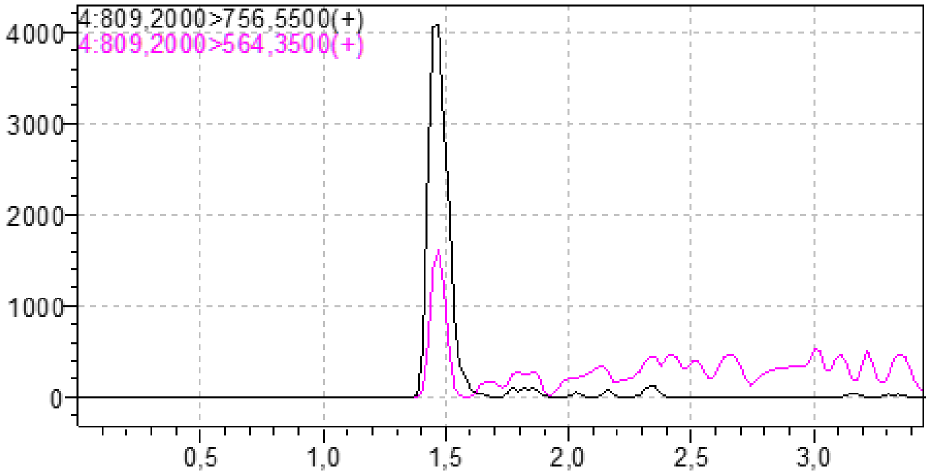

(3)**

Figure S1. Development and optimization of the matrix purification procedure. Justification for the use of double-step purification consisted of the protein precipitation and liquid-liquid extraction step. In the image (**top**) below, three experiments and their results are presented: (1) the chosen method with the highest measured peak area, (2) double-step procedure with hexane (3) liquid-liquid extraction without protein precipitation step. Corresponding chromatograms are presented below **(1-3).*** tacrolimus reference standard (99.1% purity)** internal standard ascomycin

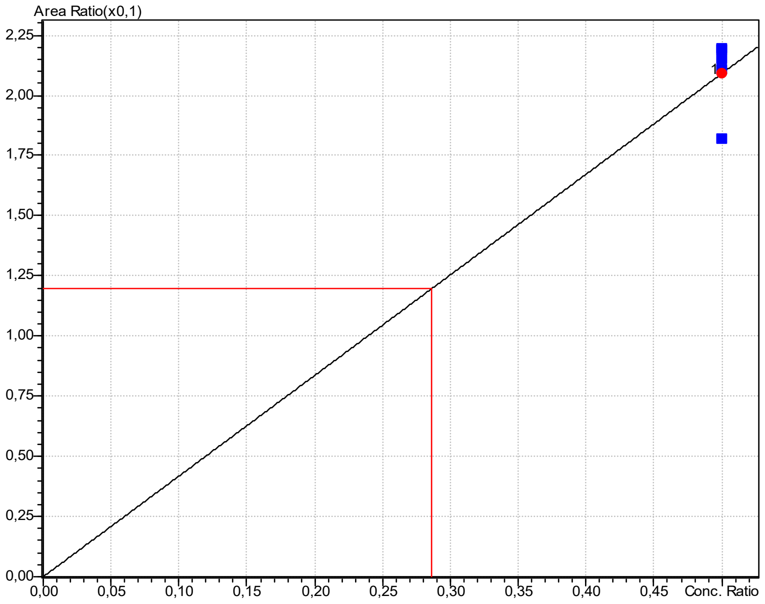

Figure S2. A single-point calibration experiment.

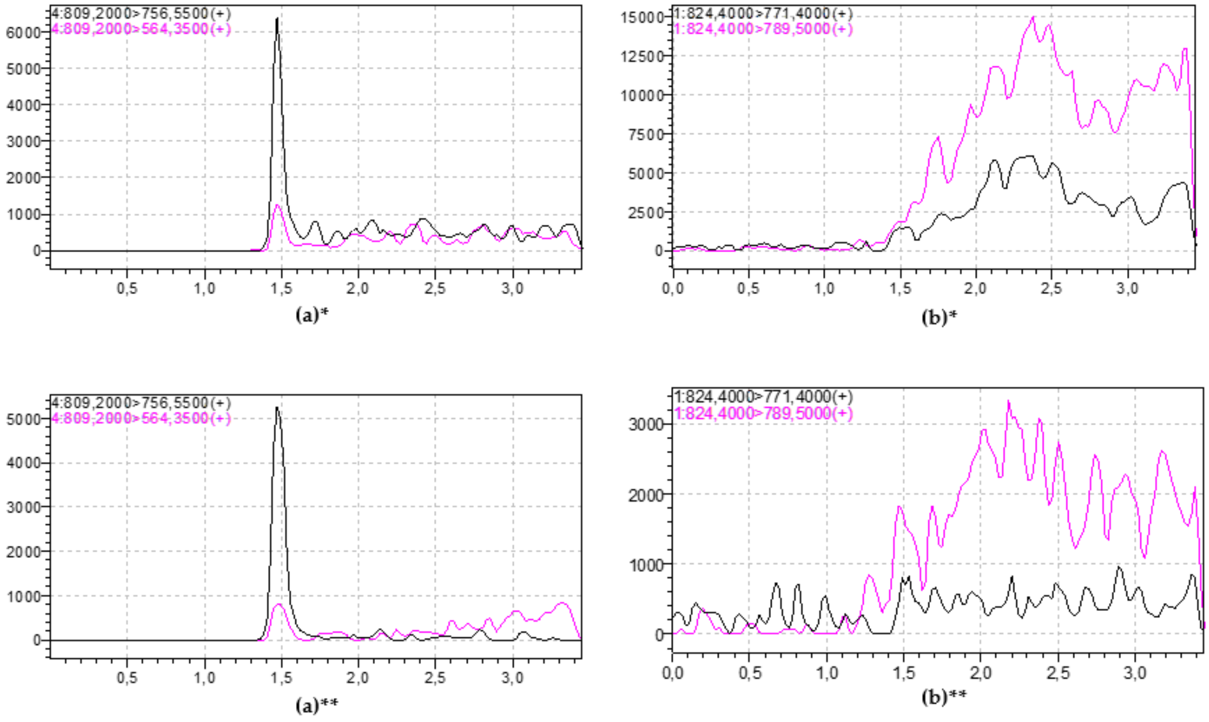

Figure S3. The comparison between LC-MS/MS chromatograms obtained using two different internal standards, ascomycin (ASC) and deuterated and ^{13}C-labeled tacrolimus (TAC^{13}C,D_2); **(a)** sample at the lower limit of quantification (0.1 pg/mL); **(b)** sample at 1.5 pg/mL. Samples with internal standard TAC^{13}C,D_2 presented substantially worse chromatographic performance than those with ASC. * internal standard ascomycin ** internal standard deuterated and ^{13}C-labeled tacrolimus.

Table S1. A single-point calibration experiment. To assess the sensitivity of the apparatus and perform a linearity experiment, twelve solutions with concentrations of 0.1 pg/mL (n=6) and 0.5 pg/mL (n=6) were produced and measured. Accuracy ranged between 87.0% and 130.2%.

Sample	Level	Red. Time	Area	Conc. [pg/mL]	Std. Conc.	Accuracy (%)	Cal. Point	S/N	ISTD Area	Area Ratio
1 (0.5 pg/mL)	1	1.509	7 860	0.52281	0.5	104.6	1	21.86	35 953	0.218628
2 (0.5 pg/mL)	1	1.496	11 976	0.51354	0.5	102.7	1	40.3	55 765	0.214753
3 (0.5 pg/mL)	1	1.494	6 569	0.50419	0.5	100.8	1	63.69	31 154	0.210844
4 (0.5 pg/mL)	1	1.493	6 126	0.52423	0.5	104.8	1	32.96	27 945	0.219224
5 (0.5 pg/mL)	1	1.497	9 770	0.43522	0.5	87	1	40.46	53 679	0.182002
6 (0.5 pg/mL)	1	1.476	11 905	0.87078	0.5	174.2	1	133.27	55 954	0.212765
1 (0.1 pg/mL)	1	1.482	13 905	0.13015	0.1	130.2	1	56.12	129 557	0.107327
2 (0.1 pg/mL)	1	1.477	9 634	0.12184	0.1	121.8	1	57.51	95 883	0.100476
3 (0.1 pg/mL)	1	1.472	2 620	0.12591	0.1	125.9	1	52.19	25 231	0.103833
4 (0.1 pg/mL)	1	1.475	3 069	0.11537	0.1	115.4	1	117.14	32 258	0.0951365
5 (0.1 pg/mL)	1	1.475	3 549	0.10035	0.1	100.3	1	39.62	42 887	0.0827518
6 (0.1 pg/mL)	1	1.482	10 905	0.10015	0.1	100.2	1	56.12	129 557	0.107327

Table S2. Justification for narrowing the linearity range. The comparison between the results obtained using the linearity range of 0.1-20 pg/mL and the range of 1-200 pg/mL in 47 patient samples. All unbound tacrolimus concentrations were successfully measured in the linearity range of 0.1-20 pg/mL, whereas only 11 when the range of 1-200 pg/mL was used. Because of the extremely low tacrolimus concentrations in plasma ultrafiltrate: 1) A substantial part of the measurements was beyond the linearity range of 1-200 pg/mL 2) Concentration that were measured successfully differed significantly when the range of 1-200 pg/mL was used, which is probably caused by the linearity line deviation in wider linearity range.

**Sample**	Linearity range [pg/mL]	
	0.1-20.0	1-200
12a	0.29115	-0.67403
12b	0.38647	-1.2509
12c	1.20518	-0.69428
12d	0.82271	-1.04342
13a	2.41368	-0.01878
13b	1.24141	-0.40868
13c	2.25	0.23951
13d	7.3403	5.93255
14a	0.2603	-1.40834
14b	0.24327	-1.92795
14c	0.2583	-1.50623
14d	0.25719	-1.53539
15a	0.92421	-0.07743
15b	2.26067	0.74931
15c	0.93228	-0.00563
15d	1.03432	-0.68165
16a	0.66977	-1.27855
16c	1.08711	-0.74398
16d	1.23104	-0.6584
17a	0.72569	-0.1987
17b	0.9146	-1.47641
17c	0.50165	-1.13481
17d	0.16628	-1.65885
18a	0.12691	-1.7486
18b	0.27507	-1.4384
18c	0.50042	-0.70062
18d	0.34865	-1.3452
19a	1.75779	0.71906
19b	0.17569	-1.04784
19c	0.37902	-1.12299
19d	0.97668	-0.6018
20a	0.43313	-0.55187
20b	1.90394	0.38479
20c	0.28453	-1.3041
20d	2.76904	2.78308
21a	1.69152	0.29942
21b	2.70929	0.80969
21c	2.28304	0.46518
21d	1.20716	-0.19421
22a	0.15484	-1.32186
22b	0.98094	-0.68088
22c	2.26872	0.32398

22d	0.81893	-0.98513
23a	2.63071	1.18705
23b	1.32311	-0.77113
23c	0.86593	-0.74362
23d	1.48932	-0.2914
